# Advances in the treatment of glioma-related signaling pathways and mechanisms by metformin

**DOI:** 10.3389/fonc.2025.1482050

**Published:** 2025-01-29

**Authors:** Xingyuan Ma, Chao Sun, Xiao Ding, Yuhang Zhang, Tingzhen Deng, Yatao Wang, Haijun Yang, Ruiwen Ding, Haotian Li, Dawen Wang, Maohua Zheng

**Affiliations:** ^1^ The First School of Clinical Medicine, Lanzhou University, Lanzhou, China; ^2^ Department of Neurosurgery, The First Hospital of Lanzhou University, Lanzhou, China; ^3^ Department of Neurosurgery, Beijing Tiantan Hospital, Capital Medical University, Beijing, China; ^4^ The third Department of Surgery, Armed Police Hospital of Tianjin, Tianjin, China

**Keywords:** metformin, glioma, ferroptosis, oxidative stress, AMPK signaling pathway, apoptosis, glioma immunity, CLIC1

## Abstract

Metformin (MET) is a commonly used drug for the treatment of type 2 diabetes in the department of endocrinology. In recent years, due to the few clinically effective treatment options including glioma, some scholars have proposed the possibility of metformin in the treatment of glioma, and studies have shown that metformin has a certain inhibitory effect on this tumor. This review explores the multiple mechanisms through which metformin exerts its antitumor effects, focusing on signaling pathways such as AMPK/mTOR, ferroptosis, autophagy, apoptosis and chloride ion channels (CLIC1). Metformin’s inhibition of glioma proliferation involves complex cellular processes, including mitochondrial dysfunction, increased reactive oxygen species (ROS) production, and modulation of immune responses. Additionally, metformin affects glioma stem cells by inhibiting key pathways, including STAT3, mTOR, and AKT, and altering the tumor microenvironment. While preclinical studies suggest that metformin enhances radiosensitivity and reduces tumor recurrence, its clinical application remains in early stages, with further studies needed to optimize dosing regimens and understand its full therapeutic potential. This review provides a comprehensive analysis of metformin’s molecular mechanisms in glioma treatment and highlights its potential as a novel therapeutic strategy, especially for treatment-resistant gliomas.

## 1 Introduction

Glioma is mainly produced by glial cell carcinoma of the brain and spinal cord, which is a common primary intracranial tumor in clinical practice, accounting for about 40% of nervous system tumors. Not only that, its evil degree is high, accounting for about 80% of malignant tumors of the nervous system. Although surgical treatment and chemoradiotherapy for glioma have made certain progress in current medicine, the median survival time of these patients after diagnosis is still only about 12 months, and the main reasons affecting prognosis are the difficulty of radical resection of glioma and the tolerance to radiotherapy and chemotherapy (1, 2). Retrospective analyses of glioma patient data from international studies have demonstrated that treatment with Metformin (MET) in diabetic patients with glioblastoma (GBM) is associated with prolonged progression-free survival (3–5). In addition, the results of studies on the treatment of glioma patients by metformin found that the use of metformin in the treatment of diabetic patients with glioma has a trend of improving survival (6, 7). More scholars have found that Glioma patients with type 2 diabetes who receive metformin treatment have better prognoses and survival rates than glioma patients who do not receive metformin treatment. Therefore, metformin has certain research value in the treatment of glioma patients (7). There are two reasons why the use of metformin in diabetic patients with glioma can improve the prognosis of glioma patients. The first reason is the direct effects of metformin on glioma cells. Previous study demonstrated that metformin directly inhibits glioma cell proliferation through mechanisms such as the activation of the AMP-activated protein kinase(AMPK)/mammalian target of rapamycin(mTOR) signaling pathway, which disrupts cellular metabolism, induces autophagy, and promotes apoptosis in glioma cells (8). These effects are independent of metformin’s role in glucose regulation, suggesting a direct antitumor action. The second reason is the indirect effect of metformin through diabetes management. Metformin is widely used to manage diabetes by improving insulin sensitivity and lowering blood glucose levels. Elevated blood glucose and insulin levels have been associated with worse outcomes in cancer patients, as hyperinsulinemia can promote tumor growth (9). By effectively controlling hyperglycemia and insulin levels, metformin may indirectly improve the prognosis in diabetic glioma patients by limiting cancer-promoting conditions. This is supported by several studies that link better glycemic control to improved cancer outcomes (10, 11).

Increased lactate secretion, inhibition of mitochondrial intima complex activity, activation of AMPK/mTOR signaling pathway and induction of ferroptosis are considered to be the main mechanisms of metformin’s anticancer effects (12). Increased lactate secretion indirectly activates the AMPK/mTOR signaling pathway, eventually disrupting the REDOX(reduction-oxidation) balance and eventually leading to cell death, this process is associated with reduced cell proliferation, cell cycle arrest, autophagy, apoptosis and other forms of cell death (13, 14).

An animal experiment observed the changes of metformin concentration in different brain regions, cerebrospinal fluid, and plasma after single and multiple oral administration of metformin in rats (15), suggesting that metformin can quickly cross the blood-brain barrier after intestinal absorption and accumulate in the central nervous system, providing an experimental basis for patients taking metformin orally for anti-glioma treatment. However, a retrospective cohort study found that under normal therapeutic dose, metformin did not significantly improve the survival of glioma patients (16, 17). However, most of the anti-glioma treatment of metformin is limited to basic experiments, and few studies have applied metformin to clinical trials alone.

As mentioned earlier, metformin inhibits tumor cell proliferation, but the mechanism of action leading to this effect has not been fully identified. This article will review the mechanism of action of MET against glioma, to provide a new feasible idea for the treatment of glioma.

## 2 Metformin inhibited the proliferation of glioma cells via various process

### 2.1 Ferroptosis

The specific mechanism by which metformin induces ferroptosis to inhibit the proliferation of glioma cells has not been fully clarified. At present, most scholars tend to approach this process from the perspective of ferroptosis. Ferroptosis is a form of programmed cell death driven by iron-dependent lipid peroxidation, characterized by abnormal accumulation of iron and lipid peroxides (lipid ROS) in cells, disrupting REDOX balance and ultimately leading to cell death, mainly caused by loss of glutathione peroxidase (GPX4) activity in cells (18–21). However, in some cases, such as under the influence of drugs such as metformin, this balance can be upset. Metformin can mainly inhibit GPX4 protein expression in glioma cells, promote the expression of Long-chain acyl-CoA synthetase 4 (ACSL-4) protein, which leads to the increase of Reactive Oxygen Species(ROS) level, triggers ferroptosis, and inhibits glioma proliferation (20–23). As previously mentioned, metformin can promote ROS accumulation in tumor cells. While the relationships between metformin-induced ROS production and ferroptosis still require clarification.

A study on the mechanism by which metformin-induced ferroptosis inhibits the proliferation of breast cancer cells found that metformin can increase intracellular Fe2+ and lipid ROS levels, and can also induce ferroptosis in breast cancer cells by up-regulating miR-324-3p to inhibit GPX4 expression. This further confirmed that metformin may inhibit glioma cell proliferation by inducing ferroptosis (24). Additionally, studies have shown that in breast cancer, metformin induces ferroptosis by inhibiting autophagy via lncRNA H19 (25). Metformin has also been found to induce ferroptosis in other types of cancers; for instance, in liver cancer cells, metformin triggers ferroptosis through the p62-Keap1-Nrf2 pathway (26). Another study revealed that in lung cancer cell lines, metformin treatment increased levels of ROS and iron ions while reducing GPX4 and GSH levels; adding a ferroptosis inhibitor reversed these reductions (27). Additionally, experimental results indicate that metformin also induces ferroptosis in lung cancer cells through the Nrf2/HO-1 signaling axis (27). An international experimental study examining the impact of NDUFA4—a key component of the respiratory chain-oxidative phosphorylation pathway—on head and neck paraganglioma found that knocking down NDUFA4 enhanced metformin-induced ferroptosis, thereby strengthening the inhibitory effect of metformin on a head and neck paraganglioma mouse model (28). In an international clinical trial, metformin was used as an adjuvant therapy for glioma patients, with results showing that metformin could improve overall survival and progression-free survival, generally with good tolerability (8).

**Figure 1:**
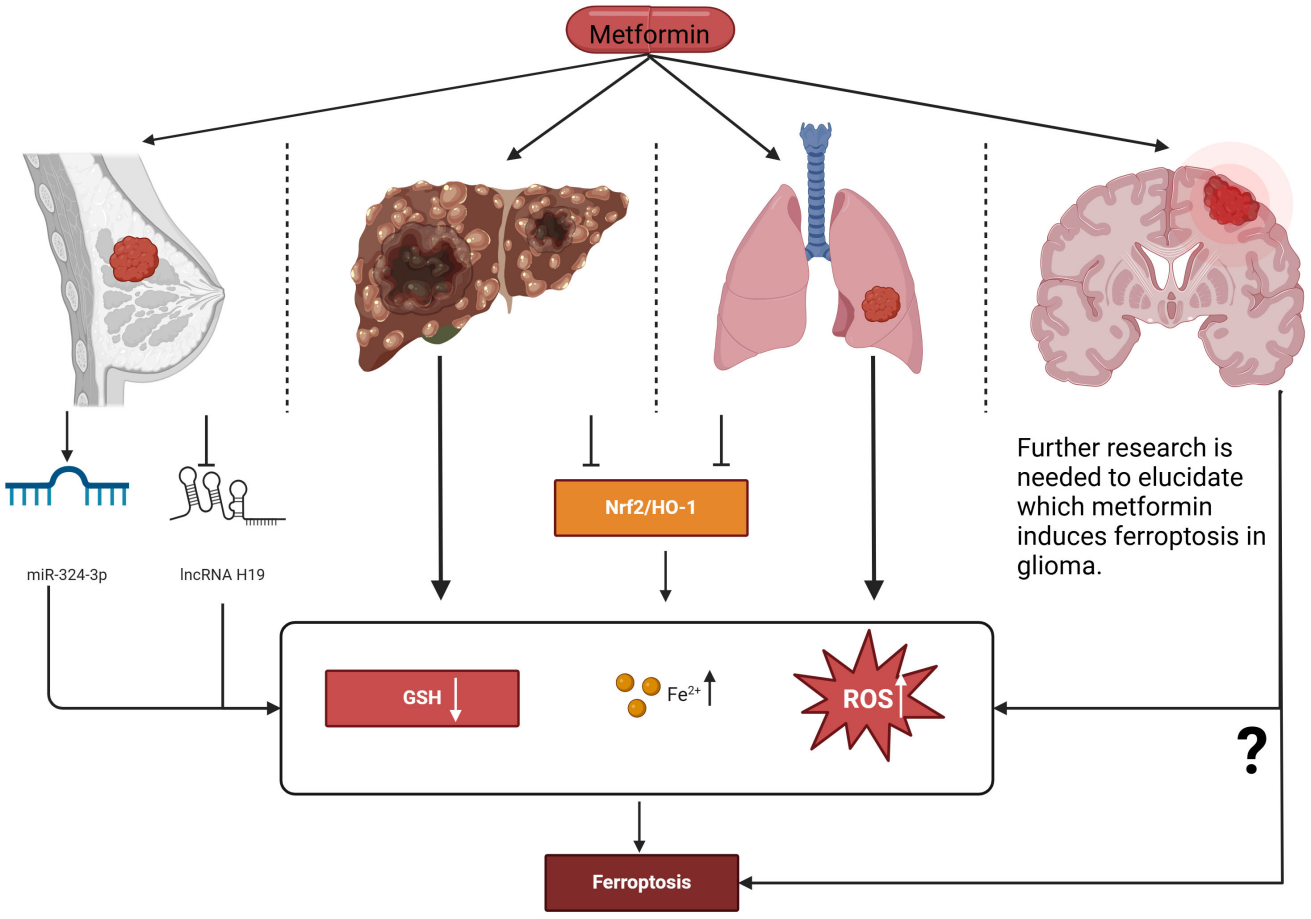
Proposed actions of metformin in inducing ferroptosis to intervene in the proliferation of various cancer cell types. Metformin induces ferroptosis in breast cancer cells by up-regulating miR-324-3p, increasing intracellular Fe²⁺ and lipid ROS levels while reducing GSH. Additionally, it can induce ferroptosis by inhibiting lncRNA H119. In both liver cancer and lung cancer, metformin induces ferroptosis through the Nrf2/HO-1 signaling axis. Further research is needed to elucidate how metformin induces ferroptosis in glioma. Nrf2, Nuclear factor erythroid 2–related factor 2; HO-1, Heme oxygenase-1; GSH, Glutathione; ROS, Reactive oxygen species.

Currently, there are few studies on metformin-induced ferroptosis to inhibit glioma cell proliferation. Although the above experimental and clinical studies have demonstrated the feasibility of metformin in treating gliomas, including suppressing cell proliferation and extending survival, these findings provide new strategies and directions for metformin’s application in glioma treatment. However, further research is needed to elucidate its specific mechanisms of action and clinical applications (**Figure 1**).

### 2.2 Autophagy

Metformin’s relationship with autophagy is multifaceted, particularly in the context of glioma. Primarily, it is known that metformin can induce autophagy as a protective response against cellular stress, and numerous studies have reported that this drug enhances autophagy, thereby affecting various cancer types including gliomas.

In glioblastoma, for instance, metformin has been observed to confer an increase in autophagy, particularly through the activation of the AMPK pathway. This is notable because AMPK activation not only promotes autophagy but also contributes to apoptotic processes, leading to a reduction in tumor cell viability. Several studies have demonstrated that metformin’s stimulation of autophagy can result in increased apoptosis in cancer cells (29, 30). For example, in hepatocellular carcinoma, metformin has been shown to activate autophagy through AMPK, which is linked to enhanced pro-apoptotic signaling (31). However, the role of autophagy in cancer treatment is not solely beneficial. ​In gliomas, autophagy can also act as a double-edged sword, providing a survival advantage to cancer stem cells and contributing to treatment resistance (32).​ Specifically, autophagy may facilitate the survival of glioma stem-like cells, which can lead to therapeutic failures (33). These cells often exhibit increased autophagic activity in response to metformin, utilizing this process to resist apoptosis induced by the drug. This adaptive mechanism can present a significant challenge in the therapeutic management of glioblastoma, necessitating a nuanced approach to treatment where modulation of autophagy may enhance the efficacy of metformin.

Moreover, metformin’s interactions with autophagy in glioma can be further supported by evidence showing that the drug, in combination with other therapies, can alter the tumor microenvironment (34). For instance, co-targeting autophagy along with immunotherapy has been shown to enhance the immune response against glioma (35), suggesting that careful modulation of autophagy could potentially improve treatment outcomes. By strategically leveraging both the apoptotic and autophagic pathways, it may be possible to develop more effective treatment strategies against glioma that can overcome the prevalent issue of drug resistance. Considering the complexity of metformin’s impact on autophagy, further exploration is warranted. Understanding how to balance its autophagic effects could improve therapeutic strategies and outcomes for patients suffering from glioma.

### 2.3 Necrosis

Research has increasingly focused on the role of necrosis, especially necroptosis, in metformin’s therapeutic efficacy against glioma cells (36). Necroptosis is a regulated form of necrosis that can be activated by stress or damage signals, distinguishing it from traditional forms of cell death like apoptosis and autophagy. Metformin has been found to exacerbate glucose deprivation-induced cell death, characterized by energetic and oxidative stress that glioma cells experience when glucose is limited (37). This finding is crucial as it underscores the unique mechanism of metformin, wherein it induces sustained energy stress, thereby driving cells toward necroptosis.

Furthermore, the combination of metformin with 2-deoxyglucose has demonstrated remarkable efficacy in promoting necrotic cell death in glioma cells (8). This combinatorial approach exploits the metabolic vulnerabilities of cancer cells, leading to heightened cellular damage. In a comparative context, the necrotic pathway activated by metformin is seen as complementary to its effects on apoptosis and autophagy, fortifying its overall antitumor efficacy (38). Additionally, the therapeutic implications of necroptosis in glioma treatment suggest potential pathways for enhancing metformin’s effectiveness. By further elucidating the molecular mechanisms behind necroptosis, researchers could devise novel strategies to maximize the therapeutic benefits of metformin in glioma therapy, aiming at harnessing its capacity to induce necrotic cell death effectively. This expanding understanding of necroptosis may pave the way for future clinical applications that leverage metformin’s unique action in targeting glioma cell metabolism and improving patient outcomes.

Research has started to unveil the role of necrosis, particularly necroptosis, in metformin’s therapeutic efficacy. Necroptosis is a regulated form of necrosis that can be triggered by stress or damage signals. Studies indicate that metformin treatment results in necrotic cell death, particularly in cancer cells treated with combined therapies, due to its ability to induce sustained energy stress and subsequent cellular damage. This necrotic pathway, akin to its effects on apoptosis and autophagy, contributes to metformin’s antitumor efficacy.

### 2.4 Other metformin-related pathways

In addition to ferroptosis, autophagy, and necrosis, metformin may influence other signaling pathways that lead to cell death such as apoptosis. Title 4 will elaborate on how metformin activates the AMPK/mTOR signaling pathway to inhibit glioma formation. Metformin activates signaling pathways related to mitogen-activated protein kinase (MAPK), which plays a crucial role in determining cellular responses to growth and stress signals (39, 40). The activation of MAPK pathways can lead to increased apoptosis in various cancer cells, including glioma. An early study reported that the activation of the MAPK signaling pathway could inhibit glioma cell growth and induce cell death, amplifying the antitumor effects of metformin (41). By enhancing insulin sensitivity, metformin has been shown to influence downstream insulin signaling pathways that impact cell proliferation and survival. Increased insulin sensitivity can lead to reduced levels of circulating insulin, which is linked to the activation of phosphoinositide 3-kinase (PI3K) signaling pathways (42). Dysregulation of this pathway is associated with many cancers, including glioma, as it promotes cell survival and growth. Moreover, one study suggested that metformin’s ability to decrease insulin signaling contributes to its pro-apoptotic effects, making it a potential agent against glioma cell proliferation (41).

Furthermore, metformin may exert its effects through the inhibition of the Akt/mTOR pathway, which is known to regulate autophagy and apoptosis. By downregulating this pathway, metformin can promote cell death in glioma cells, enhancing the efficacy of other therapeutic approaches, such as chemotherapy and radiation. Together, these signaling pathways elucidate the multifaceted roles of metformin in glioma treatment, highlighting its potential as a therapeutic agent that not only induces cell death but also modifies key regulatory mechanisms within the cancer cells.

## 3 Metformin acts on mitochondria to regulate oxidative stress

One of the mechanisms of action of metformin against glioma is to affect the production of ATP and regulate cellular oxygen consumption (43–46). By inhibiting the activity of mitochondrial electron transport chain I(;ETCI)and reducing mitochondrial respiration, metformin can alleviate the radiation resistance expressed by breast cancer cells after hypoxia and improve the efficiency of radiotherapy (24). A study investigating the effects of the AMPK inhibitor Compound C on the proliferation and viability of the U251 glioma cell line confirmed that glioma cell death induced by pharmacological activation of AMPK is mediated by reactive oxygen species (ROS) (47, 48). This finding aligns with the role of oxidative stress in activating pro-apoptotic signals and blocking survival pathways in glioma cells (48, 49). Additionally, in the experiments conducted by A. Isakovic et al., metformin led to significant induction of caspase-dependent apoptosis in C6 rat glioma cells, associated with c-Jun N-terminal kinase (JNK) activation, mitochondrial depolarization, and oxidative stress (49). Metformin can also inhibit cell activity by affecting the activity of superoxide dismutase (SOD) (50). There are other studies that have shown that metformin can reduce the content of SOD activity in GBM cells, resulting in a higher probability of GBM cells being exposed to oxygen-free radicals, suggesting that the inhibitory effect of metformin on GBM may be related to oxidative stress (51). Additionally, a study using metabolic profiling and stable isotope tracer analysis (SITA) investigated the impact of metformin treatment on tumor cell metabolism. The results showed that metformin inhibits the flow of carbon into the Tricarboxylic Acid (TCA) cycle, thereby affecting mitochondrial-dependent biosynthetic pathways, including citrate-dependent *de novo* lipogenesis. The study found that tumor cells with mutations in the mitochondrial ETC or those growing under hypoxia are resistant to metformin treatment; these cells continue to proliferate at metformin concentrations that would normally suppress tumor cell growth (52).

In summary, metformin has demonstrated inhibitory effects on glioma through various mechanisms, including regulating cellular oxygen consumption, affecting mitochondrial respiration, inducing AMPK activation, and regulating oxidative stress, etc. These findings provide a theoretical and experimental basis for the application of metformin in the treatment of glioma.

## 4 Metformin activates the AMPK/mTOR signaling pathway to inhibit glioma formation

The study on the activation of the AMPK/mTOR signaling pathway by metformin to inhibit glioma formation has achieved a series of important results at home and abroad. These studies have explored the inhibitory effect of metformin on glioma and its potential mechanism from molecular mechanism, and cell experiments to clinical trials.

It has been reported that metformin may inhibit the proliferation, invasion, and migration of cancer cells by regulating the AMPK/mTOR pathway (53, 54). During the development of glioma, metformin leads to the phosphorylation of acetyl-CoA carboxylase by activating the AMPK signaling pathway, resulting in the blocking of mTOR and inhibiting the growth of glioma. Gao et al. proved that metformin led to increased expression of AMPK and decreased expression of mTOR protein, thus inhibiting proliferation and increasing autophagy of tumor cells (31). Studies have shown that one of the anti-cancer effects of metformin is to change the balance between AMPK activation and mTOR inhibition, resulting in the negative regulation of HIF-1α (34). In addition, a foreign *in vitro* experimental study on human glioma A172 cells found that metformin increased the expression of AMPK/pAMPK/Bax protein and decreased the expression of mTOR/Bcl-2 protein in a dose and time-dependent manner, thereby inhibiting the proliferation and apoptosis of A172 cells. Reduce the invasion and migration ability of A172 cells (55). All these confirmed the role of the AMPK/mTOR signaling pathway in metformin anti-glioma, suggesting its potential application prospects.

Moreover, some Chinese scholars have found that dysfunction of the mTOR signaling pathway is closely related to tumor formation, and mTOR protein is highly expressed in glioma, suggesting that abnormal mTOR signaling may be an important pathogenesis of glioma. The carcinogenic activity of the mTOR signaling pathway is mainly activated by 4EBP1 and S6K1 proteins (56, 57). mTOR is negatively regulated by AMPK, and AMPK inhibits mTOR activity by indirectly regulating TSC2 and TSC1 together to form tumor suppressor complexes. This study discussed the changes of metformin on 4EBP1, S6K1, and mTOR in colon cancer cells from the mRNA level and protein level, and confirmed that metformin can further promote cell apoptosis by inhibiting the activity of the mTOR signaling pathway and inhibiting the levels of 4EBP1 and S6K1, showing an obvious dose-dependent effect (58).

Experiments on animal brain tumor models have demonstrated the synergistic effect between radiotherapy and ketogenic diet, and the activation of AMPK and inhibition of mTOR by metformin have anticancer activity *in vitro*. They then conducted a single-institution Phase I clinical trial of MET combination therapy in 13 patients receiving radiation therapy, and the median progression-free survival of newly diagnosed and relapsed disease was 10 and 4 months, respectively, suggesting the feasibility of metformin intervention in glioma treatment (59).

Based on the above Chinese and international research findings, it can be concluded that metformin inhibits the proliferation and diffusion of glioma cells by stimulating the AMPK/mTOR signaling pathway, and this mechanism has been verified in experiments and clinical trials. Metformin has shown some efficacy and safety in the treatment of glioma as an adjunct therapy, but further studies are needed to determine the optimal treatment regimen and dosage. At the same time, the combination of metformin with other chemotherapy agents or radiotherapy may enhance its anti-tumor effect and provide more options for glioma treatment.

## 5 Metformin promotes apoptosis of glioma cells

Metformin has recently gained attention for its potential antitumor effects, particularly in various cancer types, including glioblastoma (34). Its capacity to induce apoptosis in cancer cells is attributed to the activation of the AMPK pathway. This activation leads to the inhibition of the mTOR signaling pathway, which plays a crucial role in regulating cell growth and survival. The inhibition of mTOR results in the suppression of anti-apoptotic proteins, promoting cell death. In addition to inhibiting anti-apoptotic factors, metformin influences the expression of pro-apoptotic proteins (60). Notably, it upregulates proteins such as Bax while downregulating Bcl-2, which further facilitates apoptotic processes. This modulation of apoptotic signaling pathways indicates metformin’s potential as an effective cancer treatment (60). In addition, other mechanisms such as cell cycle arrest, mitochondria-dependent apoptosis pathway, inhibition of tumor cell metabolism, oxidative stress and DNA damage also exist. The following are detailed descriptions of other mechanisms.

Cell cycle arrest: Metformin is also able to block glioma cells in specific cell cycle phases, such as G0/G1 or G2/M, by affecting the expression of cell cycle regulatory proteins. Domestic studies usually use cell and molecular biology experimental methods to explore the effects of metformin on the glioma cell cycle and apoptosis, and the research results show that metformin can significantly change the cycle distribution of glioma cells (61, 62). At the same time, metformin can also induce apoptosis of glioma cells, which is related to the activation of the mitochondrial pathway and death receptor pathway. The mechanism by which metformin leads to apoptosis through cell cycle arrest can be explained as follows: when cells are blocked at specific cycle stages, they are unable to complete the normal process of DNA replication and division, resulting in increased intracellular pressure and accumulation of DNA damage, and these changes ultimately trigger the cell’s apoptosis program (63).

Mitochondria-dependent apoptosis pathway: metformin can induce the loss of mitochondrial membrane potential and release apoptosis-related factors such as cytochrome C, thereby activating the caspase cascade and ultimately leading to apoptosis (64, 65). This process is a key step in the mitochondria-dependent apoptosis pathway. Some researchers have explored the effects of metformin on mitochondria-dependent apoptosis pathways through cellular and molecular biology experiments (58, 59). They found that metformin-induced mitochondrial dysfunction in glioma cells, including a decline in mitochondrial membrane potential and the release of cytochrome C. These changes further activated the caspase cascade, leading to the occurrence of apoptosis. At the same time, another Chinese study also found that metformin can regulate the expression of Bcl-2 family proteins, which are key regulatory factors in mitochondria-dependent apoptosis pathways. By regulating the balance of Bcl-2 family proteins, metformin promotes apoptosis through the mitochondrial pathway (49, 66).

Inhibition of tumor cell metabolism: Metformin can reduce intracellular glucose levels and insulin signaling, thereby inhibiting the glycolysis process of tumor cells to further weaken the growth and proliferation of glioma cells. In a Chinese study, researchers found that in addition to glycolysis, metformin can also interfere with fatty acid synthesis amino acid metabolism, and other processes in tumor cells, which are also crucial for the growth and survival of tumor cells. By inhibiting these metabolic pathways, metformin further increases the apoptosis rate of tumor cells (67).

Oxidative stress and DNA damage: A study investigating metformin’s role as an adjuvant in glioblastoma therapy utilized three glioblastoma cell lines: U87MG, LNZ308, and LN229. The cells were treated with metformin, and various cellular functions were assessed, including proliferation, apoptosis, migration, and epithelial-mesenchymal transition (EMT) status (68). Furthermore, Gene Set Enrichment Analysis (GSEA) was employed to evaluate the molecular changes in the transcriptome of treated cells. The analysis revealed significant alterations in pathways related to apoptosis and metabolic processes. Reactive Oxygen Species (ROS) levels were measured using DCFH-DA and MitoSOX Red, showing that metformin increased oxidative stress within the cells (69). This elevation of ROS levels is linked to apoptosis and suggests that metformin may induce cell death through oxidative mechanisms. Mitochondrial dynamics were assessed using JC-1 dye and Western blotting, which examined mitochondrial membrane potential and biogenesis (70). Metformin treatment was found to disrupt mitochondrial function, further contributing to reduced cell viability (71). The combination of metformin with TMZ, a standard treatment for glioblastoma, produced varied effects across different cell lines. Notably, in TMZ-resistant LN229 cells, metformin suppressed key proteins, including mitochondrial transcription factor A, Twist, and O-6-methylguanine-DNA methyltransferase (MGMT) (33), which are associated with drug resistance and cancer cell survival. In addition, the study also revealed that metformin can directly or indirectly induce DNA damage in glioma cells. This damage may be due to metformin interfering with DNA repair mechanisms or promoting the production of DNA damage factors. DNA damage further activates the apoptosis signaling pathway of cells, such as the activation of the caspase cascade, thus promoting the apoptosis of glioma cells (49, 52, 64).

**Figure 2:**
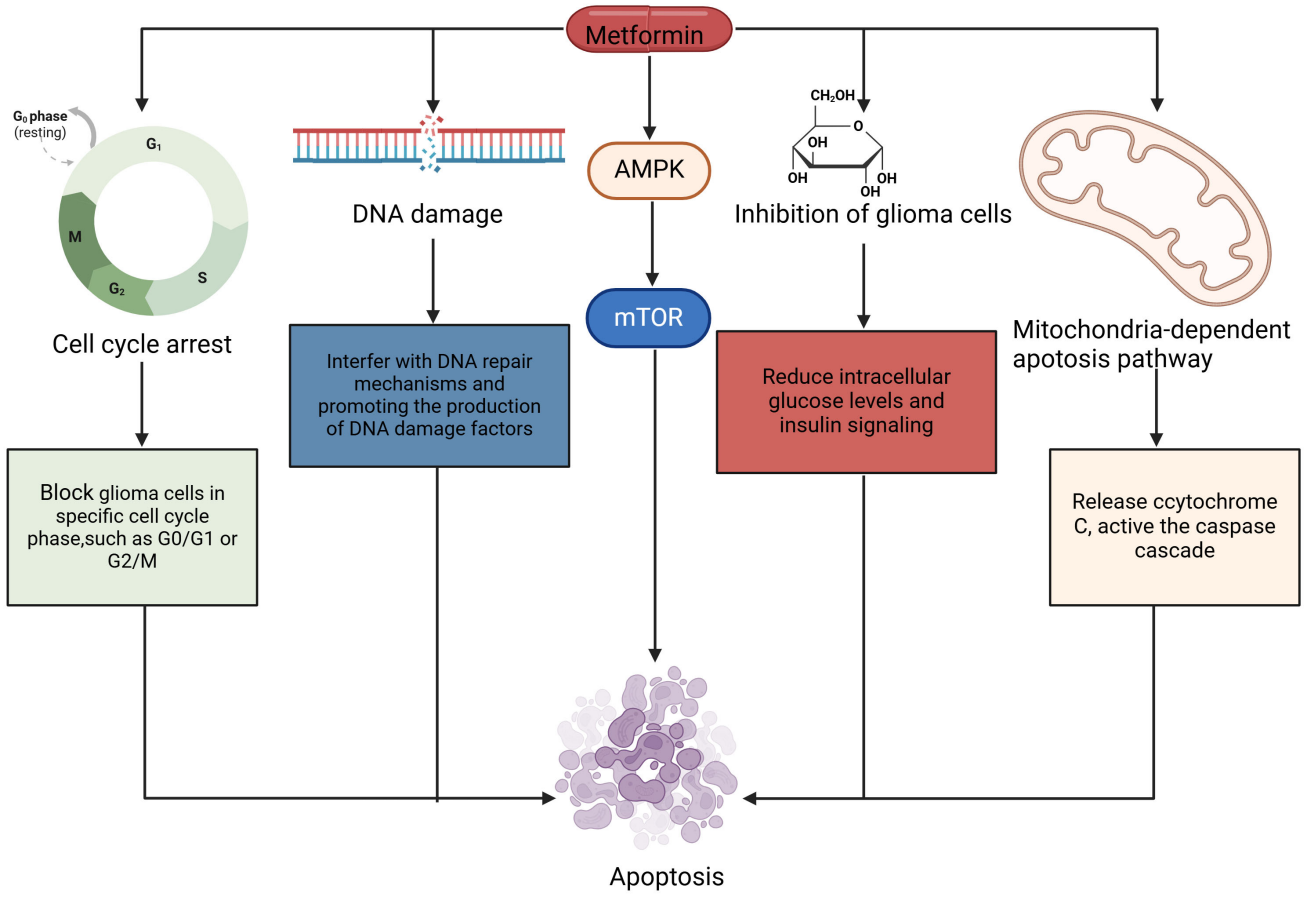
The mechanism by which metformin induces apoptosis in glioma cells. Cell cycle arrest, mitochondria-dependent apoptosis pathway, cell metabolism, and DNA damage are all involved in the apoptosis. Activated AMPK contributed to apoptosis of glioma cells. AMPK, 5’-AMP-activated Protein Kinase; mTOR, Mammalian Target of Rapamycin.

In summary, metformin disrupts the normal proliferation process of glioma cells and triggers apoptosis by activating the AMPK/mTOR signaling pathway, cell cycle arrest, mitochondria-dependent apoptosis pathway, inhibition of tumor cell metabolism, oxidative stress, and DNA damage, which provides a new idea for the treatment of glioma. Chinese and international studies have confirmed these effects of metformin through experimental methods and explored the related molecular mechanisms in depth (**Figure 2**).

## 6 Regulation of glioma immunity

Modulating the immune response of glioma by metformin is a complex process involving multiple mechanisms and biological processes. The author summarized recent detailed explanations of how metformin regulates the immune response in glioma, based on both Chinese and international studies (72–74).

Activation of AMPK signaling pathway: Metformin acts as an activator of AMPK, triggering a series of biochemical reactions that help regulate intracellular energy metabolism and immune responses. Activation of AMPK can further inhibit the mTOR signaling pathway, which is closely related to cell growth, proliferation, and immune regulation (72). By inhibiting mTOR, metformin can slow the growth rate of glioma cells and may alter the immunogenicity of tumor cells, thus making them easier to recognize and attack by the immune system (31, 72). In addition, metformin significantly increased tumor-infiltrating CD4+ T cells in mouse glioblastoma models, while reducing regulatory T (Treg) cells, with or without the combination of anti-programmed cell death 1 (PD-1) antibodies, according to a recent study (73). In addition, metformin reduces the CC motif chemokine receptor CCR8 and increases the expression of interleukin 17A (IL-17A). From a mechanistic point of view, metformin can reduce histone acetylation at the CCR8 promoter and inhibit CCR8 expression on tumor-infiltrating Treg cells by upregulating AMPK-activated Sirtuin 2 (SIRT2), thus enhancing the effectiveness of anti-PD-1 immunotherapy (73). Research by Jong-Ho Cha and colleagues has demonstrated that metformin blocks the inhibitory signaling of PD-L1, thereby enhancing cytotoxic T lymphocyte activity against cancer cells (74). This suggests that metformin has an antitumor effect by alleviating immunosuppression and promoting T cell-mediated immune response.

Induction of apoptosis: Metformin can also increase the clearance efficiency of the immune system by inducing apoptosis of glioma cells. Apoptosis is a programmed cell death process that eliminates damaged or abnormal cells, including tumor cells (49). Metformin induces apoptosis of glioma cells by triggering mitochondria-dependent apoptotic pathways and death receptor pathways. Activation of these apoptotic signaling pathways can release immunogenic molecules, such as Damage-associated molecular patterns (DAMPs), which attract and activate immune cells to further promote the immune response to glioma (75, 76).

Modulating immune-related gene expression: Metformin also affects the expression levels of multiple immune-related genes in human cells (64, 77). These genes include genes that encode pro-inflammatory factors, anti-inflammatory factors, chemokines, and so on (77). By regulating the expression of these genes, metformin can alter the interaction and signaling between glioma cells and surrounding immune cells, thereby affecting the activation and effector function of the immune system. The NF-κB signaling pathway is an important inflammatory regulatory pathway and is also closely related to the occurrence and development of tumors (78). Metformin may regulate the immune response and proliferation capacity of gliomas by influencing the activity of the NF-κB signaling pathway (78). This regulatory effect helps to enhance the immune system’s ability to monitor and clear gliomas (79).

Modulating tumor immune microenvironment Reprogramming: The tumor immune microenvironment determines whether a tumor has benign or malignant biological properties (80). This immunosuppressive microecosystem consists of tumor-associated macrophages (TAM), tumor-associated fat cells, cancer-associated fibroblasts (CAF), and certain types of lymphocytes (81, 82). In addition, bacteria-mediated metformin-supported peptide hydrogels can reprogram the tumor immune microenvironment in glioblastoma (83).

In conclusion, metformin regulates the immune response of glioma through various mechanisms, including activating the AMPK signaling pathway, regulating oxidative stress response, affecting the expression of immune-related genes, and tumor immune microenvironment. Together, these effects can inhibit the growth and proliferation of glioma cells and enhance the immune system’s ability to monitor and clear tumors. It is important to note that the regulatory effect of metformin on glioma immune response may vary depending on individual differences, drug dosage, and duration of treatment.

## 7 Inhibitory effect of metformin on glioma stem cells

An international study on the combination of drugs for glioma patients found that the combination of metformin and sorafenib could improve the therapeutic effect of GSC cell lines, and also reduce the proliferation of glioma stem cells. Verena Leidgens et al. (84) used metformin and combined drugs to intervene in GBM glioma stem cells, and observed that metformin may inhibit the specific phosphorylation of mTOR and Signal Transducer and Activator of Transcription 3(STAT3) by activating AMPK, and metformin and statins also reduced the total STAT3 level at high doses. It is suggested that metformin may be a promising new strategy to regulate the effect of STAT3 on glioma stem cells and then treat glioma. In addition, it has been suggested that metformin can affect the expression of AMPK, and then mediate the decline of AKT expression, and finally play an anti-glioma cell role by inhibiting glioma stem cells. This point was confirmed by studies by Atsushi Sato et al. (85, 86), who observed that the activation of Forkhead box O3(FOXO3) which is a transcription factor may affect the differentiation process of glioma stem cells, leading to their differentiation into non-cancer cells and playing a certain anti-glioma effect. FOXO3 activation is sufficient to induce differentiation of glioma-initiating cells with stem cell-like characteristics and inhibit their tumor-initiating potential. Here, they identified metformin (an anti-diabetic drug) as a therapeutic activator of FOXO3 (86). Metformin activates FOXO3 and promotes the differentiation of these stem cell-like glioma-initiating cells into non-tumorigenic cells (86). Furthermore, metformin enhances FOXO3 activation and differentiation through AMPK activation.

**Figure 3:**
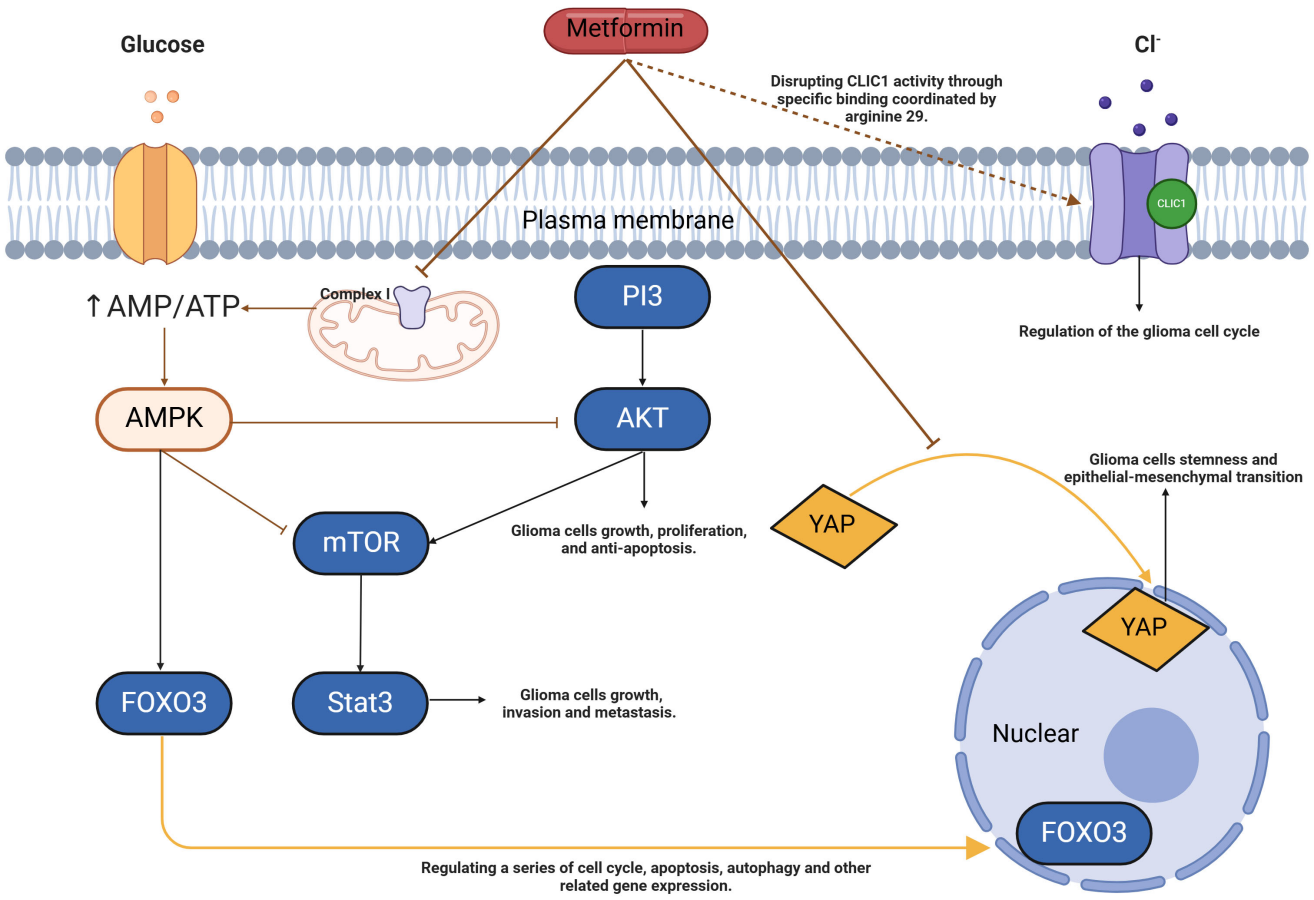
Model of metformin action in glioma stem cells. Metformin can increase the AMP/ATP ratio by inhibiting complex I of the electron transport chain, leading to changes in energy load that activate AMPK. When AMPK is activated, it further influences FOXO3, a key transcription factor. FOXO3 can translocate into the nucleus and regulate the expression of a series of genes related to cell cycle regulation, apoptosis, and autophagy. Through AMPK activation, metformin inhibits the production of mTOR and the phosphorylation of AKT and STAT3 in glioma stem cells, which suppresses tumor growth, invasion, and metastasis, thereby inducing autophagy and apoptosis. Additionally, metformin inhibits the nuclear abundance of YAP, resulting in its retention in the cytoplasm, which suppresses stemness and epithelial-mesenchymal transition in glioma cells. Metformin also interferes with intracellular chloride ion activity through specific binding at arginine 29, exerting an antiproliferative effect. AMP, Adenosine Monophosphate; ATP, Adenosine Triphosphate; AMPK, 5’-AMP-activated Protein Kinase; FOXO3, Forkhead Box O3; mTOR, Mammalian Target of Rapamycin; AKT, Protein Kinase B; STAT3, Signal Transducer and Activator of Transcription 3; YAP, Yes-associated Protein; CLIC1, Chloride Intracellular Channel 1.

At present, the literature on the inhibitory effect of metformin on glioma stem cells mainly focuses on the studies on the AMPK-mTOR/STAT3, AMPK-FOXO3 and AMPK-P13K/AKT pathways of MET (**Figure 3**).

In terms of the AMPK-mTOR/STAT3 pathway: Metformin can increase the AMP/ATP ratio in cells and activate AMPK, thus initiating a series of downstream metabolic and growth regulation reactions, such as the inhibition of mTOR (87). In glioma stem cells, overactivation of mTOR is often associated with abnormal cell proliferation and survival. In addition to directly affecting mTOR, activation of AMPK may also affect the activity of STAT3 (signal transduction and transcriptional activator 3) (84, 87). STAT3 is a key transcription factor that is abnormally activated in a variety of tumors, including gliomas, and promotes tumor growth, invasion, and metastasis. Metformin may indirectly or directly affect the phosphorylation and nuclear translocation of STAT3 through the AMPK-mTOR pathway, thereby reducing its transcriptional activity and inhibiting the malignant phenotype associated with glioma stem cells (84, 87–89). The combination of temozolomide and metformin significantly reduced secondary glial ball formation and expansion in glioma stem cells (90). Metformin effectively inhibits temozolomide-induced AKT activation, and the combination of the two drugs enhances the reduction of phosphorylation of mTOR, 4EBP1, and S6K. In addition, the combination of the two drugs was accompanied by strong AMPK activation, but the pathway was not conclusive. Temozolomide and metformin synergically inhibit the proliferation of glioma stem cells by down-regulating the AKT-mTOR signaling pathway. This combination therapy could be a promising option for patients with advanced glioblastoma.

In terms of the AMPK-FOX03 pathway: When AMPK is activated, it can further affect a variety of downstream targets, among which FOXO3 is a key transcription factor. FOXO3 is a tumor suppressor whose activity is often suppressed in a variety of tumors, including gliomas. In the activated state, FOXO3 can enter the nucleus and regulate the expression of a series of genes related to cell cycle, apoptosis, and autophagy (91). By activating AMPK, metformin promotes the nuclear translocation and transcriptional activity of FOXO3 and then inhibits the proliferation, self-renewal, and invasion ability of glioma stem cells.

AMPK-P13K/AKT pathway: The PI3K/AKT pathway is an important intracellular signal transduction pathway involved in cell growth, proliferation, survival, and migration (92). When activated, AMPK can indirectly or directly reduce the phosphorylation level of PI3K or prevent the activation of AKT to inhibit the activity of the PI3K/AKT pathway, thereby interfering with various malignant phenotypes of glioma stem cells, including proliferation, migration, invasion, and resistance to apoptosis (93). Yu et al. (93) found that Temozolomide acted synergically with metformin to inhibit the proliferation of glioma stem cells and produce the highest apoptosis rate compared with either drug alone. Moreover, it was found that temozolomide-mediated cytotoxicity of glioma stem cells may be stimulated by metformin, which has a synergistic effect and may involve the inhibition of Akt phosphorylation (93, 94).

In terms of the Hippo pathway: The transcriptional regulator YAP/TAZ acts as a downstream effector of the Hippo signaling pathway and is a key effector of the Hippo pathway (95). After Hippo signaling activation, YAP/TAZ translocates to the nucleus, where it acts as a transcriptional co-activator of other key regulatory factors (96). Transcriptional regulatory activity of YAP/TAZ plays a key role in biological development, and cell growth, and is often dysregulated during cancer progression (97). Yuan et al. (98) found that metformin inhibited the formation and size of glioma spheroids and inhibited the expression of CD133, a marker associated with glioma dryness. Mechanistically, metformin inhibits the nuclear abundance of YAP and subsequently causes its cytoplasmic retention, thereby reducing YAP transcriptional regulatory activity. Importantly, overexpression of the YAP mutant (YAP-5SA) attenuates the inhibition of metformin on the stem and epithelial-mesenchymal transformation of glioma cells.

Intracellular chloride channel 1 (CLIC1): tmCLIC1 activity controls glioblastoma proliferation. CLIC1 has been suggested as a dominant metformin receptor in glioblastoma stem cells (99). Metformin disrupts CLIC1 activity through specific binding coordinated by arginine 29. Arginine mutation No. 29 prevents metformin from binding to CLIC1, eliminating metformin’s inhibition of glioblastoma cell proliferation and metformin-dependent mitochondrial respiration in 2D and 3D models. In addition, it was demonstrated by zebrafish embryos and mouse tumor models transplanted with glioblastoma cells that metformin binding CLIC1 is essential for the antitumor effects of metformin. In addition, inhibition of CLIC1-mediated ionic currents has a selective anti-proliferative effect on human glioblastoma stem cells (100). However, it has been found that derivatives of biguanide, Q48, and Q54, are two novel CLIC1 blockers that have higher anti-proliferative efficacy than metformin *in vitro* glioma stem cell 2D cultures and 3D spheres (99).

In summary, metformin shows potential in the treatment of glioma, especially when used in combination with sorafenib, to enhance the efficacy of GSC and inhibit its proliferation. Metformin may affect the behavior of glioma stem cells by activating AMPK and inhibiting the phosphorylation of mTOR and STAT3. At the same time, metformin may also down-regulate the expression of FOXO3 and AKT, and further inhibit the proliferation and invasion ability of glioma stem cells. These findings suggest that metformin may be a novel treatment strategy for glioma by regulating multiple signaling pathways.

## 8 Conclusion

Metformin inhibits the proliferation of glioma cells through various mechanisms, such as ferroptosis, autophagy, apoptosis, and necrosis. Furthermore, the main known mechanisms of metformin in regulating the glioma signaling pathway include regulating oxidative stress, modulating glioma immunity, activating the AMPK signaling pathway, inhibiting the mTOR signaling pathway, suppressing the nuclear abundance of YAP, and interfering with the activity of intracellular chloride channels. In addition to this, there are several other possible signaling pathways and biological processes that may be affected by metformin: The MAPK/ERK signaling pathway: The MAPK/ERK signaling pathway plays an important role in biological processes such as cell proliferation, survival, and metastasis. Metformin may regulate glioma growth and metastasis by affecting the activity of the MAPK/ERK signaling pathway. Wnt/β-catenin signaling pathway: The Wnt/β-catenin signaling pathway is involved in regulating stem cell self-renewal and cell fate determination, and also plays an important role in a variety of tumors. Metformin may affect the proliferation and differentiation of glioma stem cells by affecting the Wnt/β-catenin signaling pathway. Other unknown signaling pathways: In addition to the known signaling pathways, there may be other undiscovered signaling pathways that are affected by metformin. With further research, these new signaling pathways may be revealed, further refining the understanding of metformin in the treatment of glioma. Many studies have also shown that metformin can be used as a sensitizer for glioma cell TMZ therapy and/or radiation therapy, or as a combination agent for other drugs to treat glioma. However, most studies have certain limitations, and only experimental studies are carried out in cells outside the body. Such research models cannot show the full picture of tumor cells in the human development process, so it is necessary to further improve animal experiments or observation of glioma patients in the future. In short, metformin provides a new idea for the treatment and clinical application of glioma and provides a new opportunity for the treatment and intervention of glioma disease. In summary, although multiple signaling pathways modulating the effects of metformin in glioma treatment have been identified, there are still some unknown signaling pathways that may be affected by it. Further studies could explore these unknowns signaling pathways to understand the mechanism of action of metformin in glioma treatment more comprehensively.
